# Supplementary material for: Achieving green agricultural development: Analyzing the impact of agricultural non-point source pollution on food security and the regulation effect of environmental regulation
Source: PLoS One. 2025 Jun 4;20(6):e0324899. doi: 10.1371/journal.pone.0324899 (PMC12136423; doi:10.1371/journal.pone.0324899)
Supplement: Attachment — Submitted filename: comments- Plos one.docx [file pone.0324899.s001.docx]

Manuscript Title: Achieving green agricultural development: Analyzing the impact of agricultural nonpoint source pollution on food security and the regulation effect of environmental regulation

Manuscript Id. PONE-D-24-54342

Xu and Lu reported proposed finding based on empirical data from 30 provinces in China during 2010-2022, and nicely explores how agricultural non-point source pollution affects food security and the regulatory effects of environmental regulations, The study shows that the intensification of agricultural non-point source pollution significantly inhibits the growth of food security levels, especially in western regions, major grain-producing areas, and grain-balanced regions. This study proposes that local governments ought to heighten their focus on and regulation of agricultural non-point source pollution, harmonize agricultural technology with land mobility, refine human resource allocation, and fortify environmental regulatory reforms to safeguard food security. I recommend this paper for publication after minor revisions.

**comments**

1. Authors should define green agriculture in introduction section as the green agriculture is mentioned in the title.
2. Sentence mentioned in the introduction (first page) *In 2021, the net per-acre usage of nitrogen, phosphorus, and potassium compound fertilizers for China's three major grain crops was* ***8.39 kg, 0.44 kg, 0.56 kg, and 15.54*** *kg, respectively* needs to be check again. it seems some typographical error.
3. A total 11 tables have been presented in the paper, therefore authors should present one or two table in figure format.
4. Authors should justify why Total K (potassium) and BOD (Biological Oxygen Demand) has been excluded from analysis.
